# RUNX1 regulates the proliferation and chemoresistance of colorectal cancer through the Hedgehog signaling pathway

**DOI:** 10.7150/jca.51338

**Published:** 2021-09-03

**Authors:** Qingyuan Li, Qiuhua Lai, Chengcheng He, Haonan Zhang, Xingzhu Pan, Haolin Li, Qun Yan, Yuxin Fang, Side Liu, Aimin Li

**Affiliations:** 1 Guangdong Provincial Key Laboratory of Gastroenterology, Department of Gastroenterology, Nanfang Hospital, Southern Medical University, Guangzhou, Guangdong, China; 2 The First School of Clinical Medicine, Southern Medical University, Guangzhou, Guangdong, China

**Keywords:** colorectal cancer, RUNX1, Hedgehog signaling pathway, proliferation, chemoresistance

## Abstract

**Background:** Chemoresistance is one of the main causes of recurrence in colorectal cancer (CRC) patients and leads to a poor prognosis. To characterize RUNX1 expression in colorectal cancer (CRC) and elucidate its mechanistic involvement in the tumor biology of this disease.

**Methods:** The expression of RUNX1 in CRC and normal tissues was detected by bioinformatics analysis. Cell proliferation was measured by CCK-8 and clonogenic assays. In vivo tumor progression was assessed with a xenograft mouse model. Cell drug sensitivity tests and flow cytometry were performed to analyze CRC cell chemoresistance. RUNX1, key molecules of the Hedgehog signaling pathway, and ABCG2 were detected by qRT-PCR and Western blotting.

**Results:** RUNX1 expression is upregulated in CRC tissues. RUNX1 enhanced CRC cell resistance to 5-fluorouracil (5-FU), promoted proliferation, and inhibited 5-FU-induced apoptosis. Mechanistically, RUNX1 can activate the Hedgehog signaling pathway and promote the expression of ABCG2 in CRC cells.

**Conclusions:** Our study demonstrated that RUNX1 promotes CRC proliferation and chemoresistance by activating the Hedgehog signaling pathway and ABCG2 expression.

## Background

Colorectal cancer (CRC) is one of the most common malignant tumors of the digestive tract and is a serious threat to human health, and its incidence is rising in young and middle-aged individuals^1^. 5-Fluorouracil (5-FU) is the backbone of therapy for patients with metastatic colorectal cancer (mCRC)^2^. Overexpression of the multidrug resistance (MDR) transporter ABCG2 in vitro has been shown to cause resistance to 5-FU, a component of the most commonly adopted regimens for treating CRC^3^. Abnormal proliferation and uncontrolled growth are the main characteristics of cancer, and chemotherapy failure is the major cause of recurrence and poor prognosis in CRC patients^4^. Thus, identifying the molecular mechanisms that influence CRC progression or chemotherapy is critical for improving the chemotherapy of the disease.

Runt-related transcription factor 1 (RUNX1) is a member of the RUNX family of transcription factors (RUNX1, RUNX2, RUNX3), and this family is highly conserved during evolution and plays a decisive role in encoding proteins associated with a variety of cell lineages^5^,^6^. Numerous studies have revealed that RUNX1 plays an oncogene or tumor suppressor role in a variety of solid tumors^6^-^11^. In particular, RUNX1 promotes the progression of CRC cells and plays a carcinogenic role^12^,^13^.

In previous studies, the RUNX family and Hedgehog signaling pathway were shown to be closely related. We found that RUNX1 and the Hedgehog signaling pathway are interdependent in hemogenic endothelium (HE) and hematopoietic stem and progenitor cells (HSPCs)^14^,^15^, and the Hedgehog signaling pathway can be induced directly by RUNX2 and RUNX3 in osteoblastic cells^16^,^17^. In CRC, RUNX1 has been reported to promote metastasis by activating the Wnt/β-catenin signaling pathway^12^. At the same time, more than one of the pathways is active in tumors, and these pathways are unbalanced; moreover, impaired crosstalk contributes to tumor development^18^,^19^.

In the present study, we report that RUNX1 acts as an oncogene in CRC by activating the Hedgehog signaling pathway to promote cancer cell proliferation and regulating ABCG2 expression to reduce the sensitivity of cancer chemotherapy.

## Materials and Methods

### Bioinformatics analysis

UALCAN (http://ualcan.path.uab.edu/) is an effective online analysis and mining website for in-depth analyses of gene expression data using The Cancer Genome Atlas (TCGA) RNA-sequencing (RNA-seq) and clinical data from 31 cancer types^20^. The UCSC XENA (https://xenabrowser.net/) website provides information on the 31 cancer types and clinical data from the TCGA database^21^. The biological functions of these differentially expressed RUNX1 genes were comprehensively assessed by Kyoto Encyclopedia of Genes and Genomes (KEGG) pathway analysis. All enrichment analyses were carried out by WebGestalt (http://www.webgestalt.org/)^22^. Gene Expression Profiling Interactive Analysis (GEPIA) (http://gepia.cancer-pku.cn/about.html) is a newly developed interactive web server for analyzing the RNA-seq expression data of 9,736 tumors and 8,587 normal samples from the TCGA and Genotype-Tissue Expression (GTEx) projects using a standard processing pipeline^23^.

### Cell culture, plasmid construction, lentiviral construction, and cell transfection

Three human CRC cell lines (HCT116, SW480, and RKO) were purchased from the Cell Bank of Type Culture Collection (CBTCC, Chinese Academy of Sciences, Shanghai, China) and were cultured in Dulbecco's modified Eagle medium (DMEM) (Gibco, Carlsbad, CA) supplemented with 10% fetal bovine serum (FBS; Gibco, Carlsbad, CA). Cells were maintained at 37℃ in a humidified 5% CO2 atmosphere.

### Plasmid construction, lentiviral construction, and cell transfection

RUNX1 overexpression and knockdown were performed using a lentiviral packaging system. To construct overexpressing exogenous and RNA-interfered endogenous RUNX1 cell lines, full-length RUNX1 (NM_001754) was cloned into the expression vector pLenti-EF1a-EGFP-P2A-Puro-CMV (Obio Technology, Shanghai, China) and transfected into HCT116 and RKO cell lines according to the manufacturer's instructions. Knockdown of endogenous RUNX1 was mediated by designed shRNAs (Cyagen, Guangzhou, China) that were transfected into SW480 and RKO cell lines according to the manufacturer's instructions. The RUNX1 shRNA sequences were sense 5'-CCAGGTTGCAAGATTTAAT-3' and 5'-GGCAGAAACTAGATGATCA-3', and the scramble sequence was sense 5'-CCTAAGGTTAAGTCGCCCTCG-3'. Transduced cells were selected in a medium containing puromycin (#EZ2811D376, BioFroxx, Germany) (2 μg/ml) and maintained in a medium containing puromycin (1 μg/ml).

### RNA isolation and qRT-PCR

Total RNA was extracted from cells or tissues with TRIzol reagent (TaKaRa, China). qRT-PCR was performed using the PrimeScript RT Reagent Kit (#RR035A, TaKaRa, China) and SYBR Premix Ex Taq (#RR820A, TaKaRa, Dalian, China) following the manufacturer's instructions. Our results were normalized to the expression of glyceraldehyde-3-phosphate dehydrogenase (GAPDH) or U6. The specific primers used are listed in Table S1. The qRT-PCR results were analyzed to obtain the Ct values of the amplified products, and the data were analyzed by the 2-ΔΔCt method.

### Western blotting and immunohistochemistry (IHC) analysis

We performed Western blotting according to the methods of a previous study. Protein lysates were prepared, subjected to sodium dodecyl sulfate-polyacrylamide gel electrophoresis (SDS-PAGE), transferred onto polyvinylidene difluoride (PVDF) membranes, and blotted according to standard methods using antibodies to the following: RUNX1 (#4336, CST), PTCH1 (#2468, CST), PTCH2 (#2470, CST), GLI1 (#3538, CST), Shh (#2207, CST), ABCG2 (#42078, CST) and GAPDH (60004-1-Ig, Proteintech).

IHC was performed following the manufacturer's instructions (PV-6001, ZSGB-BIO, Beijing, China) using Ki-67 (27309-1-AP, Proteintech). One independent pathologist used ImageJ software to calculate gray values for pathological scoring.

### Cell counting kit assay

Cells transfected with the appropriate plasmids were seeded into a 96-well plate at 1,000 cells per well and cultured at 37 °C and 5% CO^2^ for 6 h. For the cell counting kit-8 (CCK-8) assay, the cells were incubated with 10 μL of CCK-8 (Dojindo, Japan) for 2 h at 37°C and their density was measured at a wavelength of 450 nm using the Paradigm Detection Platform (Beckman, CA, USA). The cells were further incubated for 5 days following the CCK-8 assay.

### Colony formation assays

Colony formation assays were performed in 6-well culture plates. Cells were seeded at a density of 5×10^2^ per well. The cells were incubated at 37°C in a humidified atmosphere with 5% CO2 and the medium was replaced every 3-4 days. The colonies were counted and analyzed in about 2 weeks. The experiment was performed with at least three replicates for each cell line.

### Apoptosis assays

The cells were analyzed by FACS according to the standard protocol provided by the manufacturer (BD FACSAria II). Apoptosis was measured by using an Annexin V-PE/7-Amino-Actinomycin (7-AAD) Apoptosis Detection Kit (#559763, BD Biosciences Pharmingen, US). After treatment with 5-FU (#9648, TargetMol, China) at the indicated concentrations for 48 hours, the cells were harvested by trypsinization, washed with cold phosphate-buffered saline (PBS), and then resuspended in 1X binding buffer. Then, 5 µL of PE Annexin V and 5 µL of 7-AAD were added to each tube. The suspension was then mixed well and incubated for 15 min in the dark at room temperature (RT) (25°C). After resuspension, the samples were analyzed by flow cytometry.

### In vivo experiments

Female athymic 4- to 5-week-old Balb/c (nu/nu) mice were purchased from the Laboratory Animal Services Centre of Guangdong Province and were maintained in a specific pathogen-free facility. For the tumor growth assay, 5 × 10^6^ cells were subcutaneously injected into the right and left sides of the back of the nude mice (n =5/group). The tumor volume was calculated using the following formula: V = 0.5 × D × d2 (where V represents volume, D represents the longitudinal diameter, and d represents the latitudinal diameter).

### Statistical analysis

GraphPad Prism 7.0, SPSS 22.0, and Microsoft Excel 2016 were used for statistical analysis. The data are expressed as the mean ± standard error of the mean unless otherwise stated. The student's t-test was used to detect significance between the groups, and the χ2 test was used for measuring the data. Analysis of variance (ANOVA) was used to determine differences among three or more groups followed by post hoc analysis. Spearman's correlation analysis was performed to detect the expression correlation.

## Results

### Higher RUNX1 expression was found in colon adenocarcinoma (COAD) tissues

**Fig 1:**
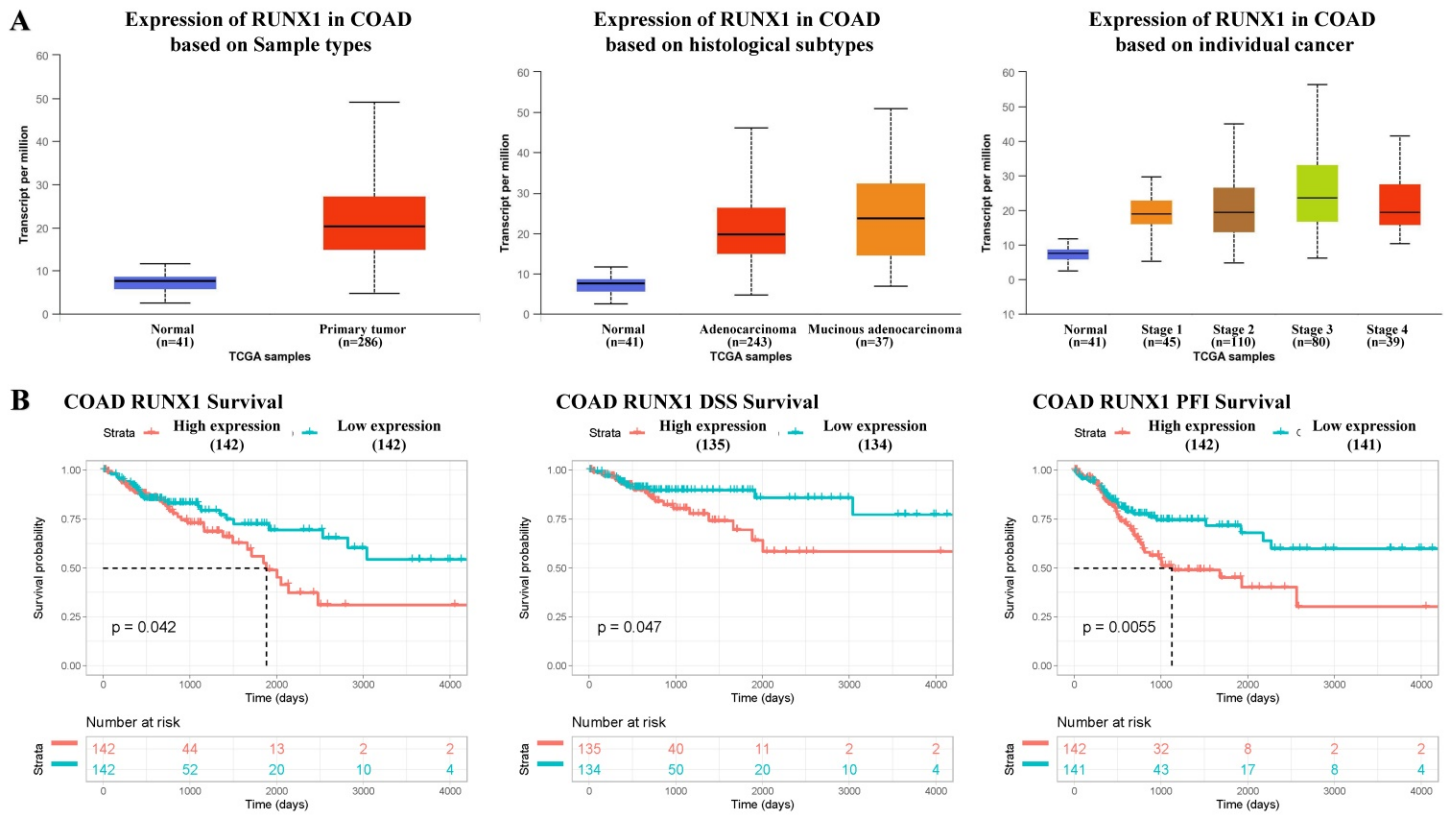
** RUNX1 expression in colorectal cancer tissues and its relationship with prognosis. A.** RUNX1 is highly expressed in the colorectal cancer and the expression of RUNX1 is increased in different histological subtypes and cancer stages (UALCAN). **B.** Percent of overall survival (OS), disease-specific survival (DSS) and platinum-free interval (PFI) survival with low RUNX1 expression was higher than that with high RUNX1 expression of colon adenocarcinoma (COAD), given by TCGA database.

According to the mRNA-seq results of the TCGA COAD dataset, RUNX1 was upregulated in COAD tissues compared with normal tissues, and the expression of RUNX1 increased to varying degrees in different histological types and cancer stages (Fig. 1A). Further, the survival analysis results suggest that high RUNX1 expression is associated with worse overall survival (OS) and disease-specific survival (DSS) and a shorter platinum-free interval (PFI) (Fig. 1B).

### RUNX1 promoted the proliferation of CRC cells in vitro

**Fig 2:**
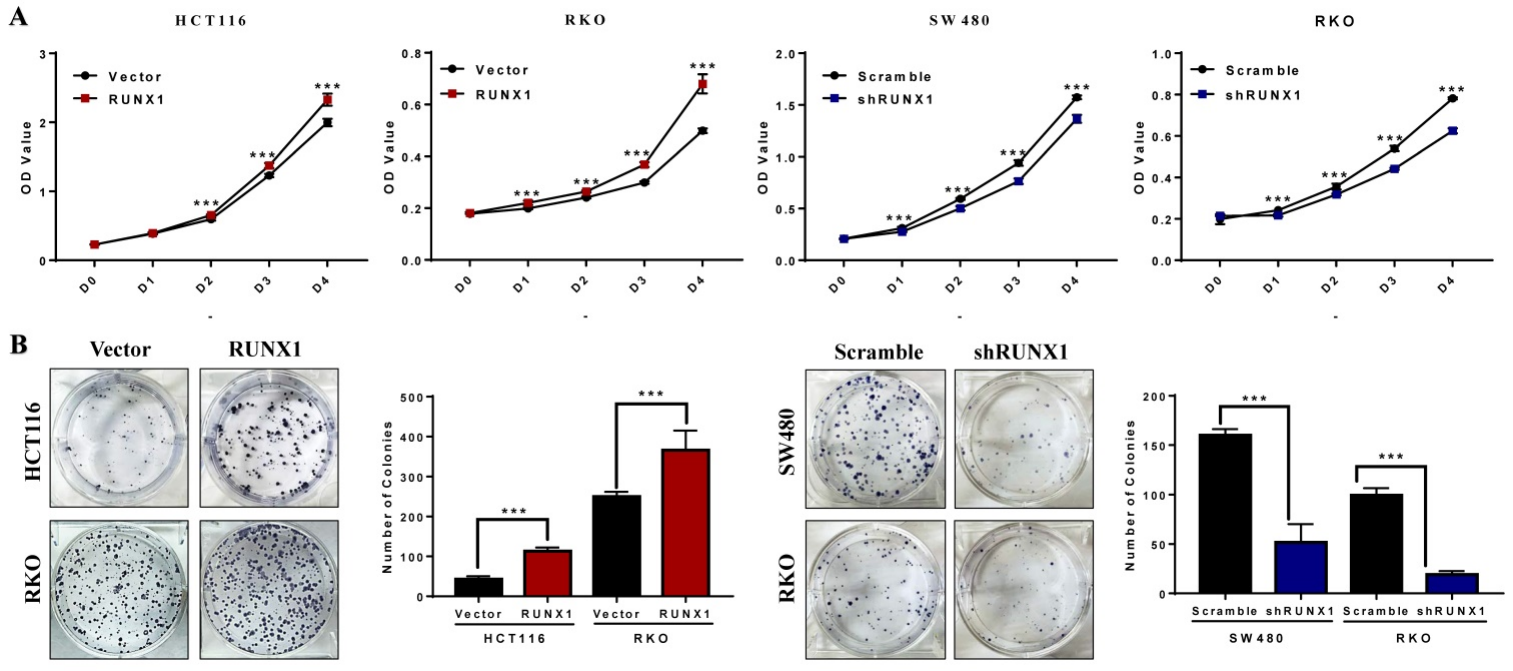
** RUNX1 promotes the proliferation of CRC in vitro. A.** Overexpressed RUNX1 stimulates cell proliferation as determined by CCK8 assay, while CRC cells with RUNX1 silencing inhibited cell proliferation. **B.** Overexpression of RUNX1 in HCT116 and RKO cells promoted CRC cells to form more colonies comparing to control cells. SW480 and RKO cells with RUNX1 silencing inhibited cells to form colonies; error bars, SD.

The CCK-8 assays demonstrated that the proliferation rates of HCT116 and RKO cells were increased when RUNX1 was overexpressed, and the proliferation rates of SW480 and RKO cells were decreased when RUNX1 was downregulated (Fig. 2A). The number of cell colonies was increased when RUNX1 was overexpressed, and the number of cell colonies was decreased when RUNX1 was downregulated (Fig. 2B).

### RUNX1 promoted the proliferation of CRC cells in vivo

**Fig 3:**
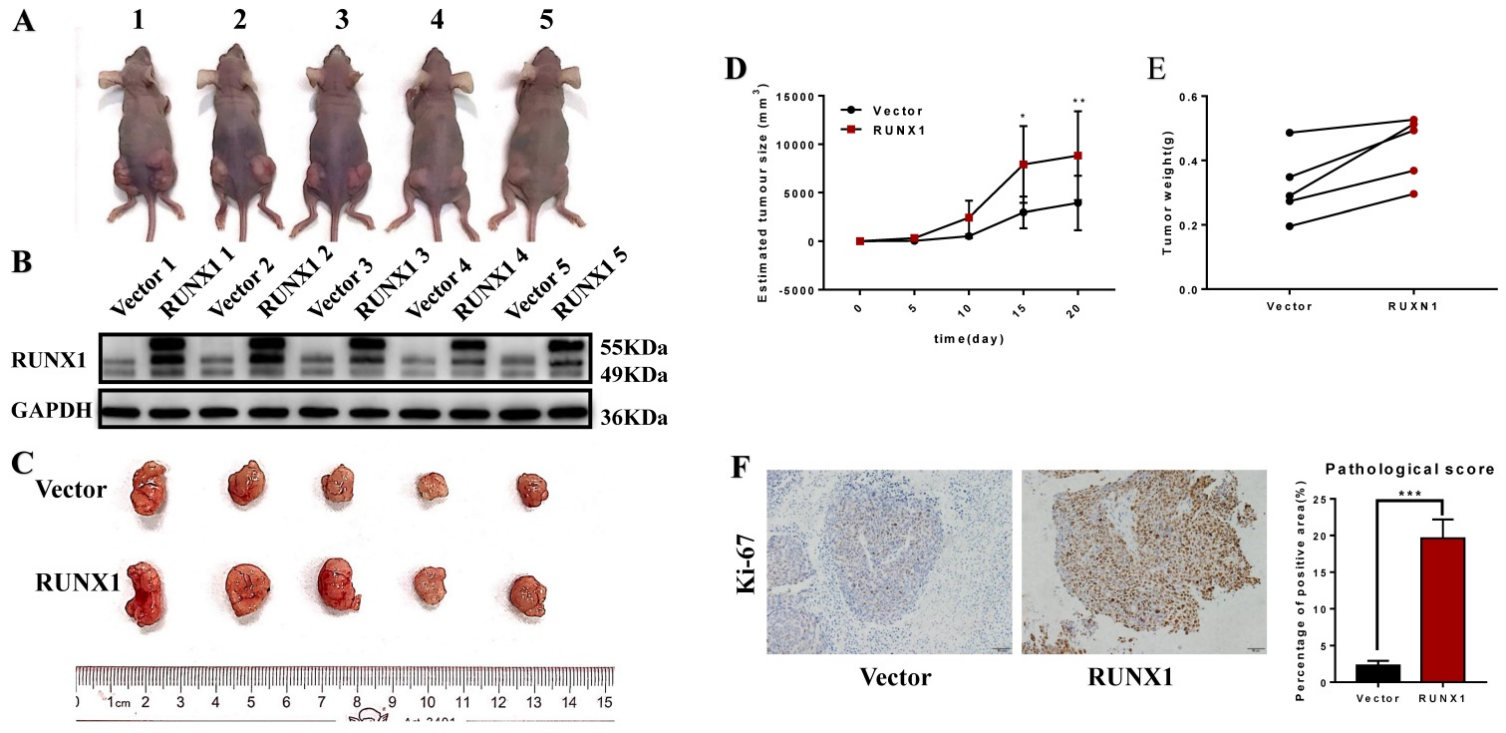
** RUNX1 promotes CRC proliferation in vivo. A.** HCT116/Vector and HCT116**/**RUNX1 cells were injected in the backside of nude mice. **B.** RUNX1 expression in the subcutaneous tumors of mice were detected by western blotting.** C.** External whole-tumors images by subcutaneous injection of HCT116/Vector and HCT116**/**RUNX1 cells were obtained. **D.** Tumor sizes were measured on the indicated days to assess the effect of RUNX1 on subcutaneous tumor growth. **E.** Tumor weight of subcutaneous tumors were obtained. **F.** IHC of Ki-67 in subcutaneous tumors were assessed. Relevant pathological scores of each were also obtained; error bars, SD.

Furthermore, we also achieved the same results in the in vivo proliferation assays. HCT116 cells stably transfected with RUNX1 and vector were injected into the right and left hips of female nude mice. We found that after 20 days, HCT116-RUNX1 promoted tumor growth compared to the control group (Fig. 3A-E). Consistent with these findings, higher Ki-67 expression levels were shown by IHC in the RUNX1-overexpressing subcutaneous tumors of the nude mice (Fig. 3F). These results strongly suggest that RUNX1 is involved in enhancing the proliferative capacity of CRC.

### RUNX1 enhances Hedgehog pathway activation in CRC

**Fig 4:**
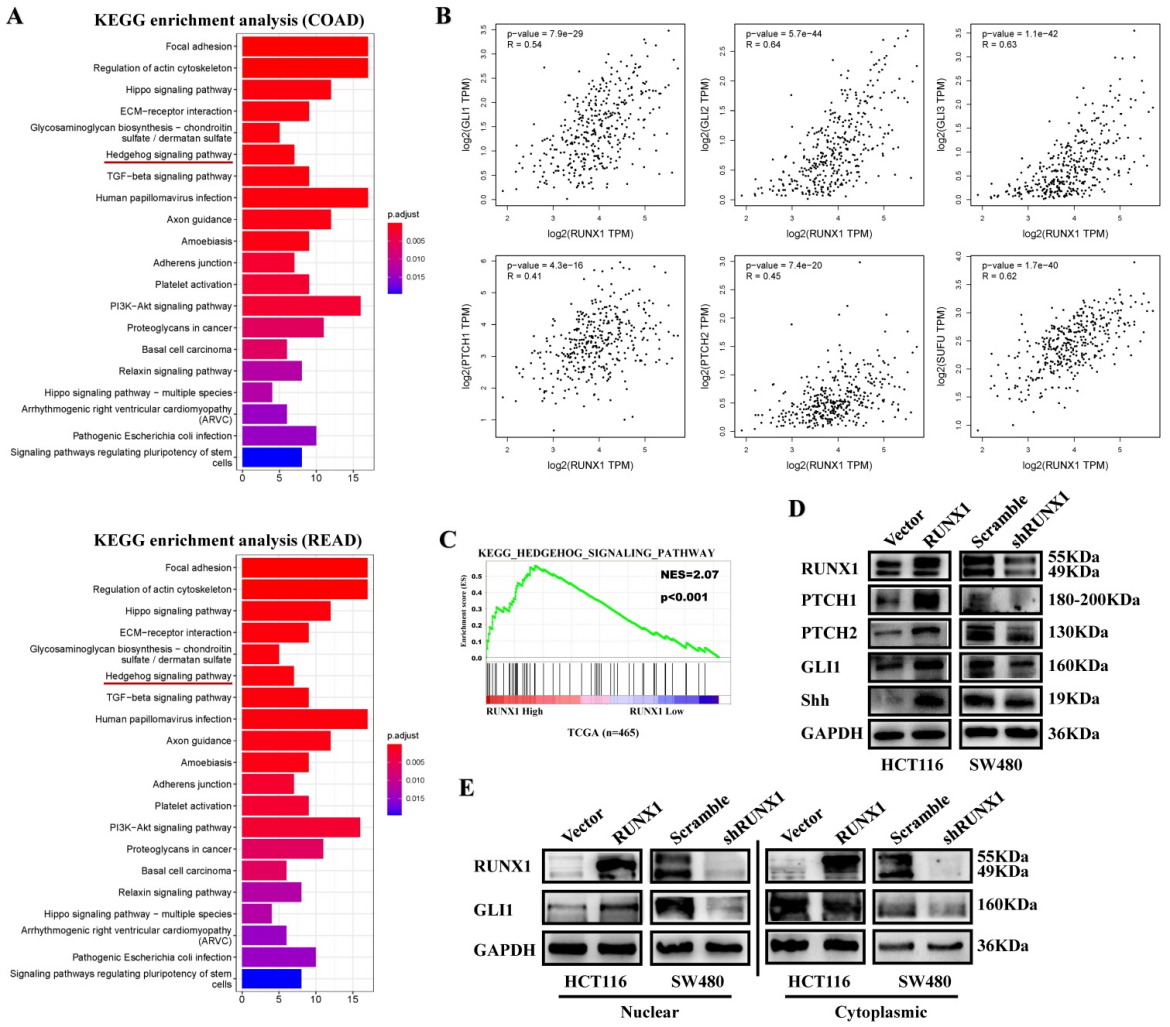
** RUNX1 promotes CRC proliferation via Hedgehog signaling pathway. A.** Enrichment of Hedgehog signaling pathway with RUNX1 expression in CRC was shown by enrichment analysis of KEGG. **B.** Relationship of RUNX1 and multiple molecules of the Hedgehog signaling pathway **C.** Enrichment of Hedgehog signaling pathway with RUNX1 expression in CRC was shown by enrichment analysis of GSEA.** D.** Expression of targeted genes activated by Hedgehog signaling pathway in HCT116/Vector, HCT116/RUNX1, SW480/scramble and SW480/shRUNX1 groups. **E.** GLI1 of the Hedgehog signaling pathway expression in HCT116/Vector and HCT116/RUNX1, SW480/scramble and SW480/shRUNX1 groups (nuclear and cytoplasmic separation assay).

To determine the potential function of RUNX1 in CRC, we analyzed the top 300 genes most similar to RUNX1 in COAD and rectum adenocarcinoma (READ) tissue samples from the TCGA database by KEGG enrichment, and the results showed that there was a correlation between RUNX and the Hedgehog signaling pathway (Fig. 4A). Through mRNA expression correlation analysis on the GEPIA website, we found that the expression of RUNX1 is positively correlated with multiple molecules of the Hedgehog pathway, such as GLI1, GLI2, GLI3, PTHC1, and PTCH2, and is statistically significant (Fig. 4B). Gene set enrichment analysis (GSEA) of the TCGA database showed that RUNX1 expression was associated with the Hedgehog signaling pathway (Fig. 4C). We used Western blotting to detect the expression of molecules in the Hedgehog pathway, and the results showed that RUNX1 increased the expression of proteins, including PTCH1, PTCH2, GLI1, and Shh, that are associated with the Hedgehog pathway (Fig. 4D). Moreover, a nuclear and cytoplasmic separation assay suggested that the expression changes of GLI1 protein were primarily shown in the nucleus (Fig. 4E).

### RUNX1 reduced the chemosensitivity of CRC cells to 5-FU

**Fig 5:**
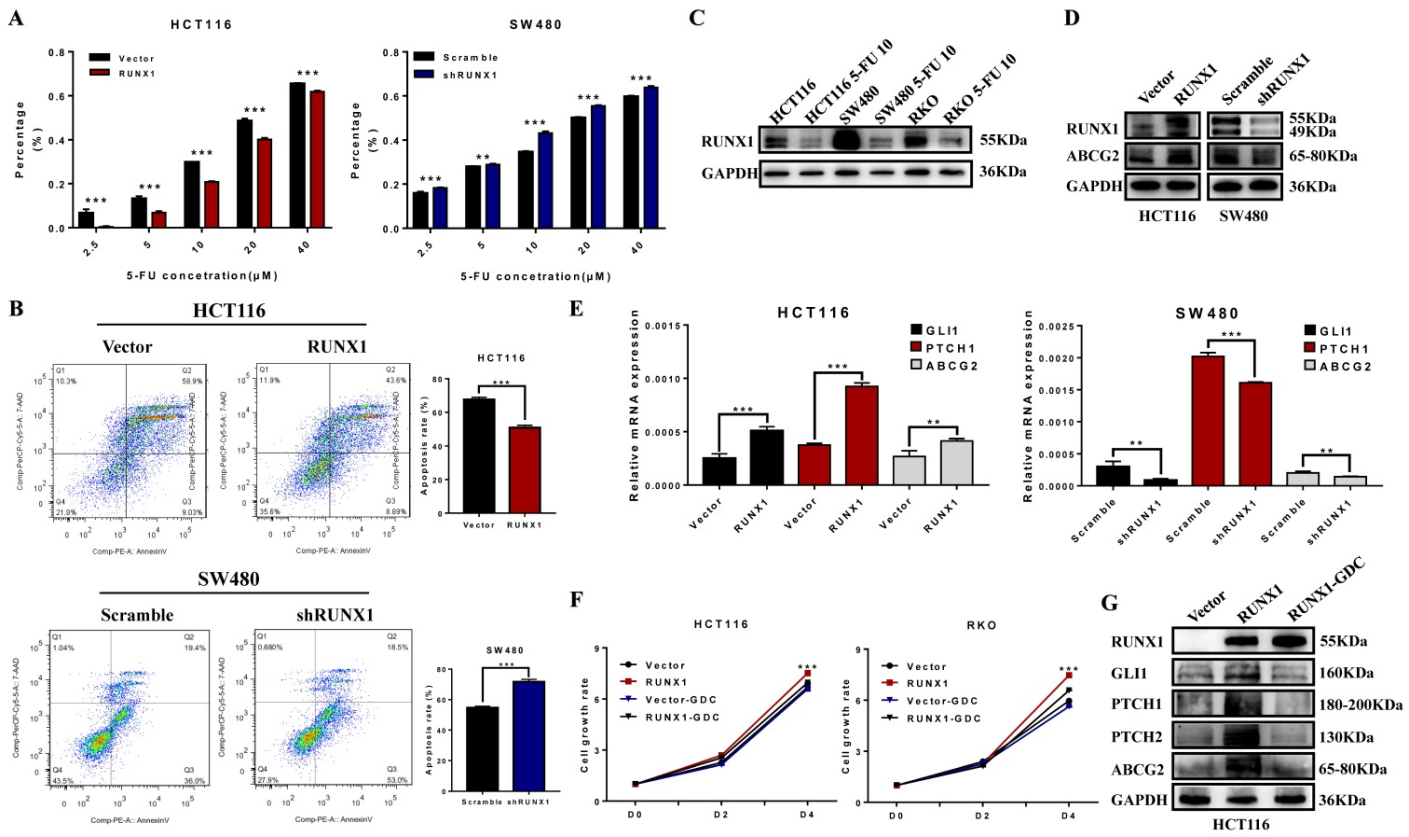
**RUNX1 decreased 5-FU sensitivity of CRC cells via Hedgehog signaling pathway. A.** 5-FU sensitivity measured by CCK8 assays in HCT116/RUNX1, SW480/shRUNX1, HCT116/Vector and SW480/Scramble cells with the different concentrations of 5-FU treated; error bars, SD.** B.** Apoptosis rate measured by flow cytometer in HCT116/RUNX1, SW480/shRUNX1, HCT116/Vector and SW480/Scramble cells with 5-FU treated; error bars, SD. **C.** Expression of RUNX1 in HCT116, SW480 and RKO cell lines with 5-FU treated. **D.** Expression of ABCG2 in HCT116/RUNX1, SW480/shRUNX1, HCT116/Vector and SW480/Scramble groups. **E.** Expression levels of GLI1 PTCH1 ABCG2 mRNA in HCT116/Vector and HCT116/RUNX1, SW480/scramble and SW480/shRUNX1 group; error bars, SD. **F.** HCT116 and RKO cell proliferation determined by CCK8 assay in Vector and RUNX1, vector with GDC and RUNX1 with GDC groups **G.** Expression of ABCG2 and targeted genes activated by Hedgehog signaling pathway in HCT116/Vector, HCT116/RUNX1, HCT116/RUNX1 with GDC groups.

To explore the possible role of RUNX1 in chemotherapy resistance, the cell growth inhibition rate was detected after treatment with a concentration gradient of 5-FU, and the results demonstrated that RUNX1 weakened the cytostatic action of 5-FU (Fig. 5A). The cell apoptosis rate was determined by an apoptosis kit and flow cytometry. The apoptosis rate was increased in the RUNX1 knockdown group, while it was dramatically decreased in the RUNX1-overexpressing group during treatment with 5-FU (Fig. 5B). In addition, we investigated the protein expression level of RUNX1 and found that RUNX1 was downregulated after treatment with 5-FU (10 µM) in the HCT116, SW480, and RKO cell lines (Fig. 5C). Furthermore, there was a positive correlation between the expression of RUNX1 and ABCG2 (Fig. 5D), and ABCG2 is a part of the superfamily of ATP-binding cassette (ABC) transporters. The mRNA expression level of GLI1, PTCH1, ABCG2 were significantly altered by RUNX1 upregulation. Similar results were observed in HCT116 cells with RUNX1 overexpression and SW480 cells with RUNX1 silencing (Fig. 5E). GDC-0449, a Hedgehog signaling pathway-specific inhibitor. Using CCK8 assays, we found that when cells were treated with GDC-0449, the proliferation ability of HCT116 and RKO RUNX1-overexpressing cell lines was decreased (Fig. 5F). Moreover, the results of Western blot can suggest that GDC-0449 can inhibit the expression of ABCG2 and targeted genes activated by the Hedgehog signaling pathway induced by RUNX1 (Fig. 5G).

## Discussion

It is generally believed that RUNX1 dosage is important during normal development and in the homeostasis of hematopoietic tissues, and growing experimental evidence implicates RUNX1 in crucial hallmarks of cancer progression, such as cell proliferation, epithelial-mesenchymal transition (EMT), or DNA repair^24^. In studies of CRC, RUNX1 regulates tumor metastasis by activating the Wnt/β-catenin signaling pathway and EMT. The lncRNA RNCR3 can be activated by RUNX1 and exerts its oncogenic role by regulating the miR-1301-3p/AKT1 axis^25^. However, no report has shown that RUNX1 has a role in chemoresistance.

An unresolved question is whether RUNX1 functions to promote tumor proliferation. First, in this report, we found that RUNX1 expression was upregulated in CRC tissue by analysis of the TCGA database. Next, we identified RUNX1 as an oncogene that promotes CRC cell proliferation both in vitro and in vivo, and the downregulation of RUNX1 had the opposite effect. Moreover, we found that RUNX1 promotes 5-FU chemoresistance in HCT116 and SW480 cells. Mechanistically, RUNX1 promotes CRC proliferation by activating the Hedgehog signaling pathway and regulating ABCG2 expression to promote chemoresistance.

Aberrant activation of the Hh signaling pathway is associated with tumorigenesis in various tissues, and most studies specify that the Hedgehog signaling pathway participates in the oncogenesis of CRC^26^. Some oncogenes promote the stemness and drug resistance of CRC by aberrant activation of the Hedgehog signaling pathway^27^-^30^.

In the ABC protein superfamily, human breast cancer resistance protein (BCRP, ABCG2) is the main efflux transporter, and its overexpression in cancer-resistant cell lines leads to MDR^31^. In human multiple myeloma, the expression of ABCG2 is regulated by the PI3K/Akt signaling pathway, which affects the drug resistance of cancer cells^32^,^33^. The expression of ABCG2 in non-small cell lung cancer affected the efficacy of some antitumor drugs^34^. Upregulation of ABCG2 was reported to play an important role in the chemoresistance of breast cancer cells^35^, and similar conclusions have been drawn from the study of gastric cancer^36^. ABCG2 was reported to be an essential factor for MDR in CRC^37^; however, whether RUNX1 can lead to MDR by regulating ABCG2 expression has not been confirmed. It has been suggested that by upregulating the expression of ABC transporter family members, the transcription factors inducing EMT can participate in the production of MDR^38^. Our previous studies have shown that RUNX1 is associated with EMT transcription factors in CRC^12^.

ABCG2 is the target of the Hedgehog signaling pathway. The abnormal expression of the transcription factors Gli1 and Gli2 in the Hh signaling pathway regulates the expression of ABCG2 in many cancers. The activation of the Hh signaling pathway regulates drug sensitivity by directly upregulating ABCG2 expression in diffuse large B-cell lymphoma (DLBCL)^39^, ovarian cancer^40^,^41^, and gastric cancer^42^. Combined with the results of this study, we consider that RUNX1 regulates ABCG2 by activating the Hedgehog signaling pathway.

## Conclusions

**Fig 6:**
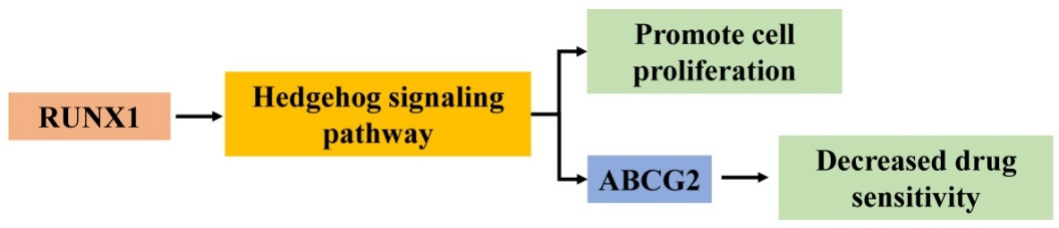
Hypothesized molecular mechanism of RUNX1 in proliferation and 5-FU sensitivity decrease of CRC via Hedgehog signaling pathway.

In summary, the current study illustrates that RUNX1 functions as an oncogene to facilitate the progression and chemotherapy resistance of CRC by enhancing the Hedgehog signaling pathway and promoting the expression of ABCG2 (Fig. 6). These findings enhance our understanding of CRC progression and chemotherapy. RUNX1 might be regarded as a potential prognostic marker and as an effective therapeutic target for CRC.

## Supplementary Material

Supplementary table.

## Abbreviations

CRC: colorectal cancer
mCRC: metastatic colorectal cancer
RUNX1: Runt-related transcription factor 1
5Fu: 5-fluorouracil
MDR: multidrug resistance
ABCG2: ATP binding cassette subfamily G member 2
HE: hemogenic endothelium
HSPCS: Hematopoietic stem and progenitor cells
KEGG: Kyoto encyclopedia of genes and genomes
TCGA: the cancer genome atlas
SDS-PAGE: sodium dodecyl sulfate-polyacrylamide gel electrophoresis
PVDF: polyvinylidene difluoride
CCK-8: cell count kit-8
COAD: colon adenocarcinoma
READ: rectum adenocarcinoma
OS: overall survival
DSS: disease-specific survival
PFI: platinum-free interval
EMT: epithelial to mesenchymal transition
