# Supplementary material for: RUNX1 regulates the proliferation and chemoresistance of colorectal cancer through the Hedgehog signaling pathway
Source: J Cancer. 2021 Sep 3;12(21):6363–71. doi: 10.7150/jca.51338 (PMC8489138; doi:10.7150/jca.51338)
Supplement: Supplementary file 1 — Supplementary table. [file jcav12p6363s1.pdf]

Table S1. Primer Sequences Used for Real-time PCR (5' to 3')

Gene	Forward primer	Reverse primer
GAPDH	GCACCGTCAAGGCTGAGAAC	TGGTGAAGACGCCAGTGGA
RUNX1	CACTGTGATGGCTGGCAATGATG	CTCTGTGGTAGGTGGCGACTTG
GLI1	AACCCTTGGAAGGTGATATGTC	TTCATACACAGATTCAGGCTCA
PTCH1	TTTTCTGCTGTTTTACAAGCCC	CATGGTAATCTGCGTTTCATGG
ABCG2	GTTCTCTTCTTCCTGACGACCAACC	GATGACACTCTGTAGTATCCGCTGATG
